# Respiratory and Allergic Health Effects of Dampness, Mold, and Dampness-Related Agents: A Review of the Epidemiologic Evidence

**DOI:** 10.1289/ehp.1002410

**Published:** 2011-01-26

**Authors:** Mark J. Mendell, Anna G. Mirer, Kerry Cheung, My Tong, Jeroen Douwes

**Affiliations:** 1 Indoor Air Quality Section, Environmental Health Laboratory Branch, California Department of Public Health, Richmond, California, USA; 2 Indoor Environment Department, Environmental Energy Technologies Division, Lawrence Berkeley National Laboratory, Berkeley, California, USA; 3 Department of Population Health Sciences, University of Wisconsin School of Medicine and Public Health, Madison, Wisconsin, USA; 4 Centre for Public Health Research, Massey University, Wellington, New Zealand

**Keywords:** allergy, asthma, dampness, fungi, indoor air, moisture, mold

## Abstract

**Objectives:**

Many studies have shown consistent associations between evident indoor dampness or mold and respiratory or allergic health effects, but causal links remain unclear. Findings on measured microbiologic factors have received little review. We conducted an updated, comprehensive review on these topics.

**Data sources:**

We reviewed eligible peer-reviewed epidemiologic studies or quantitative meta-analyses, up to late 2009, on dampness, mold, or other microbiologic agents and respiratory or allergic effects.

**Data extraction:**

We evaluated evidence for causation or association between qualitative/subjective assessments of dampness or mold (considered together) and specific health outcomes. We separately considered evidence for associations between specific quantitative measurements of microbiologic factors and each health outcome.

**Data synthesis:**

Evidence from epidemiologic studies and meta-analyses showed indoor dampness or mold to be associated consistently with increased asthma development and exacerbation, current and ever diagnosis of asthma, dyspnea, wheeze, cough, respiratory infections, bronchitis, allergic rhinitis, eczema, and upper respiratory tract symptoms. Associations were found in allergic and nonallergic individuals. Evidence strongly suggested causation of asthma exacerbation in children. Suggestive evidence was available for only a few specific measured microbiologic factors and was in part equivocal, suggesting both adverse and protective associations with health.

**Conclusions:**

Evident dampness or mold had consistent positive associations with multiple allergic and respiratory effects. Measured microbiologic agents in dust had limited suggestive associations, including both positive and negative associations for some agents. Thus, prevention and remediation of indoor dampness and mold are likely to reduce health risks, but current evidence does not support measuring specific indoor microbiologic factors to guide health-protective actions.

Dampness and mold exposures in buildings are common, with estimates ranging from 18% to 50% of buildings (Gunnbjornsdottir et al. 2006; Mudarri and Fisk 2007). A large number of studies in many geographical regions have found consistent associations between evident indoor dampness or mold and respiratory or allergic health effects in infants, children, and adults [Institute of Medicine (IOM) 2004; World Health Organization (WHO) Europe 2009]. A review by the IOM (2004) reported documented associations, but not documented causal relationships, between indoor dampness and upper respiratory tract symptoms, cough, wheeze, and asthma symptoms in sensitized persons, but not for asthma development. A more recent review by WHO up to 2007 expanded the observed associations to include asthma development, current asthma, dyspnea, and respiratory infections (WHO Europe 2009). Associations were found in both atopic and nonatopic individuals. Other published reviews or opinion pieces on this topic are available (e.g., Bornehag et al. 2004; Douwes 2005; Mudarri and Fisk 2007).

The consistent associations between evident dampness or mold and health may represent underlying causal relationships between fungal exposures and health. However, conventional quantitative measurements of fungi or other microbiologic exposures, such as counts of culturable airborne fungi, have shown less consistent associations with health effects than have qualitative assessments of visible dampness or water damage, visible mold, or mold odor. Thus, although a causal role for microbiologic exposures is plausible and likely, the evidence for this is still weak (Douwes and Pearce 2003). This is likely attributable in part to the lack of valid exposure assessment methods for the still unknown causal agents, microbial and possibly nonmicrobial, that increase with dampness and directly cause adverse respiratory and allergic effects.

Much additional epidemiologic research on qualitative and quantitative assessments of dampness and dampness-related agents has become available in the last few years. The present review combines findings of the IOM review of findings up to 2003 (IOM 2004) and a new assessment of later published studies. In this review we provide *a*) an updated, comprehensive review of available epidemiologic evidence on qualitative assessments of dampness or mold factors, and *b*) a new synthesis of evidence on quantitative measurements of microbiologic factors. Earlier work on this review (summarizing literature through 2007) was originally done to support the WHO Guidelines for Indoor Air Quality related to dampness and mold (2009).

## Methods

The online database PubMed (National Library of Medicine 2010) was searched using three groups of keywords such as dampness, damp, “water damage,” moisture, humidity, fungi, fungus, mold, mould, bacteria, or microorganisms, crossed with health, asthma, allergy, eczema, wheeze, cough, respiratory, “respiratory infection,” lung, skin, nasal, nose, “hypersensitivity pneumonitis,” alveolitis, bronchial, hypersensitivity, or inflammation and with building, house, home, residence, dwelling, office, school, or “day-care center.” A similar search was run in the ISI/Web of Knowledge database (Thomson Reuters 2010). We identified additional publications from reference lists and personal databases. Some indoor exposures/conditions were not included, for example, humidity, mattress moisture, and dust mites.

Inclusion of a primary study required the following characteristics:

- Publication in a peer-reviewed journal by November 2009
- Reporting of original data from one of the following study designs: intervention (quasi-experimental intervention), prospective (prospective cohort), retrospective (retrospective cohort or nested case–control), or cross-sectional (cross-sectional or prevalence case–control)
- No minimum study size, but if exposure was characterized only at the building level, inclusion of > 10 buildings
- Including risk factors related to dampness or microbiologic organisms/components/products, other than allergens (dust mites, cockroaches, mice)
- Including allergic or respiratory health effects
- Providing adequate control, in study design or analysis, of selection bias and confounding from key variables: sex, smoking (active in adults, passive in children), and socioeconomic status (SES; control for SES not required if SES shown not to confound in study, if adjusted for race when race highly correlated with SES, if study conducted within specific occupational groups, or if study from Nordic countries or Holland).

We gave primary consideration to associations between specific health outcomes (e.g., wheeze) and one or more qualitative assessments of indoor dampness or mold (e.g., visible dampness, visible mold, water damage, or mold odor), with the latter grouped for review. We refer to this set of factors collectively as evident dampness or mold, qualitatively assessed dampness or mold, or simply dampness or mold. Each study generally reported multiple findings (for example, four findings from a study reporting estimates for associations between visible dampness and daytime wheeze, visible dampness and nighttime wheeze, mold odor and daytime wheeze, and mold odor and nighttime wheeze). Based on all currently available evidence, including studies reviewed in the IOM report, new studies included in this review, and findings from available quantitative meta-analyses, we drew conclusions about associations between specific health outcomes and qualitatively assessed dampness or mold (excluding quantitative assessments of microbiologic factors). In “Results,” we generally refer to all ratio estimates of effect as odds ratios (ORs), although a few studies used other types of ratio estimates.

In this review we classified strength of evidence using the same categories as the IOM review on dampness and health (IOM 2004) (box ES-1, p. 8): sufficient evidence of a causal relationship, sufficient evidence of an association, limited or suggestive evidence of an association, and inadequate or insufficient evidence to determine whether an association exists. For each relationship considered, we classified the evidence using professional judgment on its persuasiveness, based on reported findings plus the strength, quality, diversity, and number of studies. Findings from quantitative meta-analyses were also considered. We placed increasing weight in the review on studies of stronger design. The strongest epidemiologic evidence was considered to come from individually randomized controlled experimental/intervention trials that added or removed risk factors. Studies considered next strongest were prospective (cohort), followed by retrospective (cohort or nested case–control), observational studies. We considered cross-sectional observational studies (including prevalence case– control studies) to provide the weakest evidence included. A set of strongly designed human studies of different designs and in different populations, with findings generally consistent in direction and magnitude, especially if magnitudes of effect were large or dose–response relations were found, was considered to provide the most persuasive overall evidence.

We drew separate conclusions, more preliminary because evidence was sparse, about associations between specific health outcomes and specific quantitatively assessed microbiologic factors. For findings on associations between a specific outcome and a specific measured indoor microbiologic factor, our criteria for evidence suggestive of associations required at least 80% consistency of estimates either ≤ 1.0 or > 1.0 (with no minimum change from the null required) among at least five estimates available from three or more studies. This rough tally of findings above or below the null did not consider magnitude of effects, precision, statistical significance, study design, or age of subjects.

## Results

### IOM review

The IOM review of epidemiologic evidence to 2003 on dampness-related health effects found no demonstrated causal associations (IOM 2004). Sufficient evidence of association was reported for four outcomes (upper respiratory tract symptoms, cough, wheeze, and asthma symptoms in sensitized persons, i.e., asthma exacerbation) for the two kinds of risk factor considered: exposure to damp indoor environments and presence of molds or other agents in damp indoor environments. Sufficient evidence of association was also reported between hypersensitivity pneumonitis in susceptible persons and mold or other agents in damp environments. The 45 studies included in the IOM review are summarized in Supplemental Material, Tables A1.1–A1.6 (doi:10.1289/ehp.1002410). Table 1 shows the numbers of studies included in the IOM review, by study design, for each type of health outcome.

### New primary research

Our literature search identified 354 articles published by late 2009 that were not included in the IOM review. Table 1 categorizes 103 studies that met the inclusion criteria. Supplemental Material, Tables A2.1–A2.16 (doi:10.1289/ehp.1002410) summarize results of these studies by 16 health outcomes. Estimated strength of association was usually reported as ORs and 95% confidence intervals (CIs), but occasionally was reported as other ratio estimates such as relative risks (RRs) or incidence rate ratios (IRRs) or as linear regression coefficients or proportions.

Considering all current evidence, most published findings involved qualitative assessments of dampness or mold, including visible water damage, visible moisture, dampness, leaks, flooding, visible condensation on windows, visible mold or mildew, and moldy or musty odor. Fewer findings were available on quantitatively measured microbiologic factors, including specific or total culturable fungi or bacteria; microscopically enumerated, noncultured fungi or bacteria; ergosterol (a structural component of fungi, used as a marker for total fungal biomass); extracellular polysaccharides (produced by fungi and used as a marker for specific fungal groups); (1→3)-β-d-glucans (a cell wall compound with immunomodulating properties found in fungi but also in some bacteria and pollens); endotoxin or lipopolysaccharide (a cell-wall compound of Gram-negative bacteria with proinflammatory properties, associated with dampness but also with many other sources); and markers of endotoxin such as 3 hydroxyl fatty acids.

### Meta-analyses

Three available quantitative meta-analyses combined multiple qualitative dampness or mold factors into a single set of dampness-related risk factors. Findings, summarized in Table 2, are described for specific outcomes below. Two meta-analyses using the same methods estimated summary ORs and 95% CIs for associations of dampness or mold in residences with respiratory effects: upper respiratory tract symptoms, cough, wheeze, asthma development, current asthma, and ever-diagnosed asthma (Fisk et al. 2007), and respiratory infections and bronchitis (Fisk et al. 2010). Antova et al. (2008) estimated summary ORs for dampness-related factors and ever-diagnosed asthma, bronchitis, allergic sensitization, hay fever, cough, and wheeze.

### Results for qualitative dampness or mold

We considered no health outcomes to have sufficient evidence to document a causal relationship with indoor dampness or mold.

We considered four health outcomes to have sufficient evidence for association with indoor dampness or mold that were already so classified in the IOM review: asthma exacerbation, cough, wheeze, and upper respiratory tract symptoms (Table 3).

For asthma exacerbation and dampness or mold, we consider current evidence sufficient to document association and strongly suggestive of causality. Among 31 currently available studies [see Supplemental Material, Tables A1.2 and A2.2 (doi:10.1289/ehp.1002410)], qualitative dampness-related factors were consistently associated with asthma exacerbation, with ORs consistently exceeding 1.0 in both adults [100% of findings in retrospective studies (ORs from 1.7 to 2.6) and 100% of findings in cross-sectional studies (ORs from 1.02 to 4.2)] and in children [100% of findings in intervention studies (protective associations, not reported as ORs), 100% of findings in prospective studies (ORs from 3.8 to 7.6), 100% of findings in retrospective studies (ORs from 1.5 to 4.9), and 95% of findings in cross-sectional studies (ORs from 1.0 to 7.6)]. Most notably, Kercsmar et al. (2006) conducted a controlled experimental intervention study on asthma exacerbation in houses of highly symptomatic asthmatic children. Comprehensive removal of dampness sources and visible mold caused dramatic reductions in asthma exacerbations. Acute care visits at 6–12 months after intervention were 90% fewer in those remediated versus controls (*p* = 0.003). This study (although of necessity unblinded), because of the implausibility of noncausal explanations for the findings and in conjunction with other available studies, strongly suggests a causal association between indoor dampness or mold and exacerbations in children with asthma.

For cough, most studies found positive associations with dampness or mold. In adults, 94% of ORs in cross-sectional studies exceeded 1.0 (range, 0.8–4.0). In children, 85% of ORs in prospective or retrospective studies exceeded 1.0 (range, 0.5–2.1), and 94% of cross-sectional ORs exceeded 1.0 (range, 0.2–5.7).

For wheeze, most studies found positive associations with dampness or mold. In adults, 100% of retrospective ORs exceeded 1.0 (range, 1.5–2.8), and 91% of cross- sectional ORs exceeded 1.0 (range, 0.4–5.8). In children, 95% of prospective or retrospective ORs exceeded 1.0 (range, 0.7–6.2), and 92% of cross-sectional ORs exceeded 1.0 (range, 0.5–8.7).

For upper respiratory tract symptoms, most studies found positive associations with dampness or mold. In adults, 81% of cross-sectional ORs exceeded 1.0 (range, 0.4–4.4). In children, 88% of prospective or retrospective ORs exceeded 1.0 (range, 1.0–1.8), and 95% of cross-sectional ORs exceeded 1.0 (range, 0.4–5.9).

We classified eight health outcomes as having sufficient evidence for association with indoor dampness or mold that were not so classified or not evaluated in the IOM review: asthma development, current asthma, asthma ever, dyspnea, respiratory infections, bronchitis, allergic rhinitis, and eczema (Table 3).

Asthma development is a health outcome of special public health importance. Five studies included in the IOM report [of the eight listed there for asthma development; see Supplemental Material, Table A1.1 (doi:10.1289/ehp.1002410)] explicitly examined associations between dampness or mold and asthma development (Jaakkola et al. 2002; Nafstad et al. 1998; Oie et al. 1999; Thorn et al. 2001; Yang et al. 1998). Eight new studies were identified (Supplemental Material, Table A2.1) (Cox-Ganser et al. 2009; Gunnbjornsdottir et al. 2006; Hyvarinen et al. 2006; Iossifova et al. 2009; Jaakkola et al. 2005; Matheson et al. 2005; Park et al. 2008; Pekkanen et al. 2007). Among all currently available studies (five studies in Supplemental Material, Table A1.1; all studies in Supplemental Material, Table A2.1), 78% of findings exceeded 1.0. In retrospective case–control studies of adults, 60% of ORs exceeded 1.0 (range, 0.8–2.2). Among children, 80% of prospective or retrospective/case–control ORs exceeded 1.0 (range, 0.6 to 4.1). The three studies in infants (Iossifova et al. 2009; Nafstad et al. 1998; Oie et al. 1999) reported ORs all exceeding 1.0 (range, 1.7–7.1); however, as asthma cannot be reliably assessed in infants, these findings should be interpreted with caution. Infant studies were excluded from the meta-analysis of Fisk et al. (2007), which reported a summary OR (95% CI) of 1.3 (0.9–2.1) for asthma development and dampness factors. One of the strongest reported studies, by Pekkanen et al. (2007), showed in an incident case–control study of asthma cases that dampness or mold in the main living area of houses was related in a dose–response fashion to asthma development in infants and children. Multivariate-adjusted ORs (95% CIs) for asthma incidence, for baseline and two increasing levels of maximum severity of moisture damage (assessed by civil engineers), were 1.0, 2.8 (1.4–5.4), and 4.0 (1.6–10.2). This well-designed study provides the strongest evidence (e.g., incident case–control, large and statistically significant effects, dose–response relation, unbiased exposure assessment), within a body of generally consistent other findings, that dampness-related exposures may cause asthma development in infants and children.

For dyspnea, considered in the IOM report to have limited or suggestive evidence of association with dampness, the number of available studies for adults and children has increased from 4 to 16, all cross-sectional except 1 controlled intervention study. The intervention study found significant improvements in perceived breathing after mold removal, fungicide application, and ventilation increase (Burr et al. 2007). Among the 14 other available studies of dampness or mold [Supplemental Material, Tables A1.3 and A2.5 (doi:10.1289/ehp.1002410)], measures of association for dampness or mold with dyspnea were predominantly (84%) > 1.0, with ORs ranging from 0.7 to 9.4 in adults and from 0.4 to 2.3 in children.

We included findings on current asthma when defined as either asthma diagnosis in prior 12 months, asthma diagnosis ever plus asthmatic symptoms in prior 12 months, or recent prescription of asthma medication. Current asthma, not specifically evaluated in the IOM review, was consistently associated in available studies [Supplemental Material, Table A2.4 (doi:10.1289/ehp.1002410)] with dampness or mold. In these cross-sectional studies of adults, children, or both, almost all ORs (94%) exceeded 1.0 (ranging from 0.3 to 13.0). Fisk et al. (2007) reported, as a summary effect estimate, an OR (95% CI) of 1.6 (1.3–1.9) for current asthma and qualitative dampness factors.

Ever-diagnosis with asthma [Supplemental Material, Table A2.3 (doi:10.1289/ehp.1002410)] was associated consistently with dampness or mold (91% of ORs; range, 0.6–2.6) in both adults and children. Most studies were cross-sectional and in children. All studies in adults and the single prospective study in children found completely consistent positive associations. Both available meta-analyses found increased summary ORs for ever-asthma diagnosis and residential dampness or mold, with ORs (95% CIs) of 1.37 (1.23–1.53) for children and adults in Fisk et al. (2007) and 1.35 (1.20–1.51) for children in Antova et al. (2008).

Studies on respiratory infections showed consistent associations between dampness or mold and respiratory infections [Supplemental Material, Table A2.11 (doi:10.1289/ehp.1002410)], including common colds, and with or without inclusion of otitis media. One cross-sectional study in adults found an elevated OR (3.1); two prospective studies of children found consistently elevated ORs (range, 1.34–5.10); and five cross-sectional studies in children found mostly (70%) elevated ORs (range, 0.65–1.85). The few findings on otitis media, in three studies in children, included ORs ranging from 1.0 to 1.37 for dampness or mold. The meta-analysis by Fisk et al. (2010) reported summary ORs and 95% CIs for various categories of respiratory infections (Table 2): for respiratory infections overall, in adults, and in children: 1.44 (1.31–1.59), 1.49 (1.14–1.95), and 1.48 (1.33–1.65), respectively, and for respiratory infections excluding nonspecific upper respiratory infections: 1.50 (1.32–1.70).

Dampness or mold was associated consistently with bronchitis [Supplemental Material, Table A2.7 (doi:10.1289/ehp.1002410)], with 96% of ORs > 1.0. Most studies were in children; the two prospective studies in children found generally increased ORs up to 3.8. Both available meta-analyses found positive associations between bronchitis and residential dampness or mold, with ORs (95% CIs) of 1.45 (1.32–1.59) for children and adults in Fisk et al. (2010) and 1.38 (1.38–1.47) for children in Antova et al. (2008).

Dampness or mold was associated consistently with allergic rhinitis (92% of findings, all in children), with ORs ranging from 0.7 to 3.5 [Supplemental Material, Table A2.14 (doi:10.1289/ehp.1002410)]. We included only allergic rhinitis outcomes defined as either medically diagnosed allergic rhinitis or the combination of rhinitis symptoms with documented atopy. The strongest single study (prospective) found dose–response increases in allergic rhinitis associated with visible mold, with ORs to 3.2 (Biagini et al. 2006).

Dampness or mold was associated consistently with eczema [Supplemental Material, Table A2.12 (doi:10.1289/ehp.1002410)], with 89% of ORs > 1.0 (range 0.2 to 2.9). The strongest study, a prospective study in children, found consistently increased ORs up to 2.9 for prenatal mold exposure to infants with no parental atopic history.

Other outcomes evaluated here but not in the prior IOM report include common cold, allergy/atopy, and altered lung function (Table 1). Common cold [Supplemental Material, Table A2.11 (doi:10.1289/ehp.1002410)] was positively associated with dampness or mold in 71% of reported findings. However, the methodologically strongest single study, a prospective study in children, found only 4 of 9 estimates elevated, with ORs ranging from 0.6 to 1.8. Therefore, we consider this association only suggestive.

Increase in allergy/atopy (excluding allergic rhinitis and eczema) [Supplemental Material, Table A2.13 (doi:10.1289/ehp.1002410)] in association with dampness or mold was found in 77% of reported assessments in the available studies; ORs ranged from 0.6 to 2.4. Findings in the strongest studies, two prospective studies in children, were overall somewhat inconsistent, as were the other studies. This association is made more plausible by the increased summary ORs in the meta-analysis by Antova et al. (2008) for sensitivity to inhaled antigens and for hay fever, as well as by the consistent association found in this review between dampness or mold and both allergic rhinitis and eczema. However, the overall evidence linking allergy/atopy and dampness or mold was inconsistent enough that we currently consider it only (strongly) suggestive.

The evidence associating altered lung function with dampness or mold [Supplemental Material, Table A2.8 (doi:10.1289/ehp.1002410)] was considered too inconsistent to draw conclusions. No eligible epidemiologic studies were found on hypersensitivity pneumonitis and dampness or mold (but see “Discussion” regarding overall evidence).

### Results for measured microbiologic factors

Findings on health risks associated with quantitatively assessed microbiologic factors were sparse across specific health outcomes and 53 specific types of microbial measurements. Suggestive associations (as defined in “Methods” for conclusions about quantitatively assessed microbiologic factors: requiring at least 80% consistency of estimates either ≤ 1.0 or > 1.0, among at least five estimates available from three or more studies) were not seen for measurements in air but were apparent for some measurements in dust (Table 4). Higher concentrations of ergosterol in dust were associated with increases in current asthma. Higher concentrations of endotoxin in dust were associated with increases in wheeze. For (1→3)-β-d-glucan in dust, although medium concentrations were associated with increases in wheeze, the highest concentrations were associated with decreases in wheeze. We consider these associations with quantitative microbiologic assessments to be only suggestive. Other microbial measurements used in reviewed studies [listed in Supplemental Material, Table A3.1 (doi:10.1289/ehp.1002410)] had inadequate or insufficient evidence to determine whether associations with specific health effects exist.

## Discussion

Epidemiologic evidence from primary studies and quantitative meta-analyses shows evident indoor dampness or mold to be associated consistently with a wide range of respiratory or allergic health effects, including asthma development and exacerbation, current and ever diagnosis of asthma, dyspnea, wheeze, cough, respiratory infections, bronchitis, allergic rhinitis, eczema, and upper respiratory tract symptoms. In addition to the consistently positive associations across many study designs, populations, ages, and health outcomes, dose–response relations with observed dampness and mold were often reported (e.g., Biagini et al. 2006; Cummings et al. 2008; Park et al. 2004; Pekkanen et al. 2007). Although available epidemiologic evidence does not yet establish that indoor dampness or mold causes human health effects, findings from one strong epidemiologic intervention study (Kercsmar et al. 2006), in conjunction with other available studies, strongly suggest causation of asthma exacerbation in children by dampness or mold. Several studies provide evidence for temporal association of dampness/mold and health effects by demonstrating increased incidence density of new asthma diagnosis among occupants of water-damaged buildings compared with periods before water damage (Cox-Ganser et al. 2005; Laney et al. 2009).

It is well accepted that hypersensitivity pneumonitis (HP), a granulomatous, cell- mediated lung inflammation, is caused by inhalation of antigens from microorganisms or other sources, although causal exposures often cannot be determined (Fink et al. 2005). Current knowledge is based on outbreak investigations and limited epidemiology, mostly in industrial and agricultural settings, but also in office buildings (Cox-Ganser et al. 2005; Kreiss 1989; Park et al. 2004) and, in both adults and children, in homes (Venkatesh and Wild 2005). One specific dampness-related mold exposure (*Trichosporon cutaneum*) is documented to cause HP in homes (Ando et al. 1995). [For more on HP, see Supplemental Material, Text A4.1 (doi:10.1289/ehp.1002410).]

Few studies included objective, replicable assessments of dampness. Both Karvonen et al. (2009) and Park et al. (2004), using scales combining area of water damage or area of water stains with subjective assessments, found exposure–response relations with multiple health outcomes. Williamson et al. (1997), using a scale based only on moisture meter readings from walls, also found positive adjusted associations, for example, ORs (95% CIs) for asthma and any dampness of 3.03 (1.65–5.57), exceeding ORs for subjective inspector-determined visible mold. Williamson et al. (1997) also found positive correlations between total moisture meter dampness score and both asthma severity (*p* = 0.0006) and predicted FEV_1_ (forced expiratory volume in 1 sec) (*p* = 0.006). One potential advantage of quantitative dampness measurements as indicators of exposure, relative to specific quantitative microbial measurements, is that they can be proxies for various dampness-related causal agents, whether microbial or chemical. Quantifying visible mold may also prove useful; however, Dales et al. (2010) found no significant relationship between measured area of visible mold and respiratory health outcomes.

Although evidence is limited that links any quantitative microbial measurements to specific health effects, in this review we have identified some preliminary associations (Table 4), all for measurements in dust: increased ergosterol with increased current asthma; increased endotoxin with increased wheeze; and for (1→3)-β-d-glucans, medium concentrations with increased wheeze but the highest concentrations with decreased wheeze. We consider these associations to be only suggestive, because of the limited number of studies, the limited factors considered in summarizing them, and the demonstrated complexity of some of these relationships, such as for endotoxin and (1→3)-β-d-glucans, each associated in multiple studies with both adverse and protective associations (Douwes et al. 2004, 2006).

Current findings thus cannot define causal microbiologic exposures or dose–response relations sufficiently to define safe levels of exposure to dampness-related agents. At present, subjectively assessed dampness or mold has the most consistently documented associations with respiratory and allergic disease. Quantifying dampness objectively has shown promise (Karvonen et al. 2009; Park et al. 2004; Williamson et al. 1997), but findings are few. For quantifying microbiologic factors, concentrations of culturable airborne organisms have fared poorly in empirical health research. Some assessments in dust, such as ergosterol as an indicator of total biomass of fungi, are more promising; others, such as endotoxin and glucans, have relationships with health too complex for simple interpretation. Polymerase chain reaction (PCR) assays for specific fungi in dust also have promise, but no studies using PCR met inclusion criteria for this review, and a standard scale now used to group PCR findings across fungi seems premature (e.g., Vesper et al. 2007). [For details, see Supplemental Material, Text A4.2 (doi:10.1289/ehp.1002410).]

Difficulties in finding clear relationships with measured microbiologic exposures may be attributable to measurement errors in exposure assessment, including measurement of noncausal factors; to effects that change with intensity and duration of exposure or age at exposure; or to interaction effects occurring with multiple exposures. Endotoxin, traditionally associated with non-dampness-related exposures such as farm animals and pets and with potential protection against atopy, has now been shown to be associated in water- damaged office buildings with observed dampness, fungal spores, and increased building-related asthmatic symptoms (Park et al. 2006; Rao et al. 2005). Adverse effects from endotoxin may be increased by other dampness-associated agents and vice versa (Park et al. 2006). In addition, moisture in buildings can increase nonbiologic emissions not measured in most dampness research, including formaldehyde [associated with increased asthma (McGwin et al. 2009; Mendell 2007)] from composite wood products (Matthews et al. 1986) and 2-ethyl-1-hexanol from moisture-related degradation of plasticizer in vinyl flooring (Norbäck et al. 2000).

Based on available evidence, dampness and mold may have enormous health and social costs worldwide. A northern European study found an 18% prevalence of indoor dampness (Gunnbjornsdottir et al. 2006). The IOM review (IOM 2004), using European and North American data, estimated that at least 20% of buildings had problems with dampness. Mudarri and Fisk (2007) estimated a 50% prevalence of dampness or fungi in U.S. houses. Fisk et al. (2007) concluded that “building dampness and mold are associated with approximately 30–50% increases in a variety of respiratory and asthma-related health outcomes.” Mudarri and Fisk (2007) estimated that 21% of current U.S. asthma cases were potentially attributable to dampness and mold in housing, for an annual national cost of $3.5 billion. Fisk et al. (2010) estimated that residential dampness or mold is associated with 8–20% of U.S. respiratory infections.

With regard to practical implications of these findings, we did not evaluate health benefits of specific strategies for remediation of dampness or mold. However, a recent expert review has concluded that the intervention of “combined elimination of moisture intrusion and leaks and removal of moldy items” had sufficient evidence of effectiveness for reducing respiratory symptoms from asthma and allergies and was ready for widespread implementation (Krieger et al. 2010).

### Limitations

Much of the epidemiology on dampness, mold, and health has used subjective reports for assessing exposure or health and thus has potential for reporting bias. Two reviews have considered whether biased subjective response by building occupants in dampness studies might have positively biased findings. On the basis of comparison of results in six studies from occupant reports versus inspector-reported dampness and clinically determined illness, Fisk et al. (2007) concluded that observed associations of respiratory health effects with dampness-related exposures were unlikely to be explained by overreporting. Bornehag et al. (2001) reported that findings of studies with independent assessment of both dampness and health effects were similar to findings of studies with more subjective information sources. Additionally, avoidance behavior (prior exposure reductions by persons with asthma) may be a source of past exposure misclassification with assessment of only current or recent exposure. However, this is not a concern in prospective or intervention studies, which have generally confirmed dampness/health associations.

Quantitative measures of exposure used in the reviewed studies also have important limitations. Measured airborne concentrations of culturable microorganisms have substantial errors, for example, from short-term estimation of airborne concentrations with large and rapid variations over time; from differential abilities of organisms to grow on specific culture media; and from nondetection by culture assays of most bioactive microbial materials, whether intact spores or fragments. Most important, culture-based or non-culture-based microbial measurements used in many studies may not target actual causal factors. All these reasons may explain the lack of consistent associations between reported microbial measurements and health. And as with glucans or endotoxins, even prior demonstration in many studies that a substance causes inflammation does not implicate it as consistently harmful, because both glucans and endotoxins have also demonstrated health-protective associations (Douwes et al. 2006; Iossifova et al. 2007). However, subjectively assessed dampness or mold has not shown protective associations, even in infants.

Finally, definitions of respiratory health effects are not standardized, potentially causing bias. In population studies, asthma is usually defined by self-reported (or parentally reported) asthma symptoms. Self-reports of doctor-diagnosed asthma are also often used. An alternative approach to questionnaires has been to use more objective measures, either alone or in combination with questionnaires. As with measures of home dampness or fungal exposures, differences in asthma definition are likely to result in differences in estimates of RRs. In addition, as mentioned above, several studies (Nafstad et al. 1998; Oie et al. 1999) focused on infants at an age where the diagnosis of asthma is uncertain. Most of these potential sources of bias are expected to underestimate any true association between indoor dampness and health effects.

The restricted scope of this review led to further limitations. The method of evaluating published evidence was largely nonquantitative. Results of available quantitative meta-analyses, however, are consistent with qualitative summaries. Publication bias in this review is likely to have inflated associations of risk factors with health effects. A formal application of available statistical methods for assessing presence of this bias was not feasible for this broad review. A search for unpublished findings, which may decrease publication bias, was not performed. Conclusions drawn from this review should thus be considered provisional until the production of quantitative summary estimates of RRs based on more thorough consideration of all available findings, with formal evaluation for publication bias.

### Evidence for plausible biologic mechanisms of health effects from dampness-related agents

Toxicologic evidence suggests plausible biologic mechanisms for the respiratory health effects associated epidemiologically with dampness or mold (WHO Europe 2009). *In vitro* and *in vivo* studies have demonstrated diverse inflammatory, cytotoxic, and immunosuppressive responses after exposure to the spores, metabolites, and components of specific microbial species found in damp buildings. Repeated immune activation and prolonged inflammation by microbiologic exposures may contribute to inflammation-related diseases such as asthma. The immunosuppressive response demonstrated in animals exposed to fungal spores associated with damp buildings may explain a link to respiratory infections.

The wide variety of health effects associated with dampness and mold cannot be explained by a single mechanism. Epidemiologic evidence suggests involvement of both allergic and nonallergic mechanisms, as both atopic and nonatopic individuals are susceptible to adverse effects of dampness or mold (e.g., Cox-Ganser et al. 2005; Dales et al. 2006; Douwes et al. 2006; Kuyucu et al. 2006). The inflammatory responses demonstrated in many microbiologic exposures include histamine release by non-immunoglobulin E–mediated mechanisms, providing plausible mechanisms for the occurrence of allergy-like symptoms in nonsensitized individuals. Increased human susceptibility to severe asthma exacerbation from fungal exposures has been demonstrated with genetic polymorphisms related to chitinase, suggesting mechanisms involving fungal chitin (Wu et al. 2010).

Some available evidence is consistent with involvement of fungal toxins in some health effects associated with damp environments, although this has been debated extensively in the literature (Bennett and Klich 2003; Jarvis and Miller 2005). Recently, animal models with curdlan (a specific triple-helical form of fungal glucan) and several toxic fungal metabolites have demonstrated inflammatory, nonallergic respiratory health effects consistent with the epidemiology of dampness (Miller et al. 2010; Rand et al. 2010). Observed synergistic interactions in toxicologic studies among microbial agents present in damp buildings, including specific fungi, actinomycetes, and amoebae (Penttinen et al. 2006; Yli-Pirila et al. 2007) suggest that immunotoxic effects of fungal and bacterial strains typically found in damp buildings may be potentiated during joint exposures. Such potentiation could explain difficulties in identifying specific causal exposures for health effects in damp buildings.

Many limitations of culture-based microbial assessments for investigating causes of dampness-related health effects have long been evident. Additional support for the need to investigate non-culture-based microbial assessment methods has been provided by the demonstration (Gorny et al. 2002) that fungi and actinomycetes can emit large numbers of airborne particles smaller than spores and not detectable by culture but with demonstrated immunogenic properties. These findings provide additional plausibility for health effects associated with microbial growth but not measurable with culture assays.

### The hygiene hypothesis

As summarized in this review, indoor dampness or mold is consistently associated with increased respiratory health risks, and microbial exposures have been suggested (but not proven) to play a causal role. On the other hand, an increasing number of studies suggest that early-life microbiologic exposures to endotoxin or specific fungal agents may protect against atopy and allergic disease. This potentially protective effect is consistent with the “hygiene hypothesis,” which postulates that growing up in a more microbiologically hygienic environment may increase the risk of developing respiratory allergies (e.g., Douwes et al. 2004, 2006; Liu and Leung 2006).

However, the evidence for protective effects of microbial exposures has not been consistent, as increased health risks have been associated with some specific measured exposures (e.g., Bolte et al. 2003; Dharmage et al. 2001; Michel et al. 1996). Some of these inconsistencies, found for endotoxin, (1→3)-β-d-glucans, and fungi, may be related to timing or dose of exposure, as has been recently hypothesized (Douwes et al. 2007), but evidence is still weak. For instance, Iossifova et al. (2007, 2009), in prospective data, identified nonmonotonic relationships between (1→3)-β-d-glucans in dust and recurrent wheeze, wheeze with atopy, and an index for future asthma: Risks increased at increasing low concentrations, reached a maximum at 60 μg/g dust, and then decreased at increasing high concentrations. Similar patterns have also been observed with dust mite antigen (Tovey et al. 2008).

At present, modest exposure to some microbial exposures under certain circumstances appears to protect against allergies and allergic asthma but not wheeze; however, as indicated previously, the overall evidence is inconsistent. Damp or moldy buildings seem only to increase, not decrease, the development of respiratory disease, both in allergic and nonallergic subjects including infants.

### Suggested research

A focused research program in this area might include *a*) studies to identify and improve objective tools and metrics that, in assessing either dampness or specific related factors (microbiologic or nonbiologic), optimally predict disease; *b*) studies to characterize dose–response relations, to determine safe levels and identify age-or dose-related protective effects; and *c*) strong studies (intervention or prospective) designed in the aggregate to document causality between dampness or mold and key health effects such as asthma or respiratory infections. Genetic epidemiology may enhance abilities to detect causal exposures and identify mechanisms (Wu et al. 2010). Indoor occupational settings and schools, with multiple advantages for study efficiency and logistics, have been underutilized. Good examples to follow include the strong disease prediction by an objective and easily interpreted tool, the electronic resistance-type moisture meter (Williamson et al. 1997), and the well-designed and extremely effective remediation study by Kercsmar et al. (2006). Although future findings will improve health-protective policies, health-protective actions need not await further etiologic research.

## Conclusion

Based on the material reviewed here, there is sufficient evidence of an association between indoor dampness-related factors and a wide range of respiratory or allergic health effects (Table 3), including asthma development, asthma exacerbation, current asthma, ever asthma, dyspnea, wheeze, cough, respiratory infections, bronchitis, allergic rhinitis, eczema, and upper respiratory tract symptoms. There is suggestive evidence of associations with health effects for several non-culture-based measurements related to fungi and bacteria in dust, although some of these associations seem equivocal. No evidence suggests protective effects of evident dampness and mold. Mechanisms seem likely to be both allergic and nonallergic. Available quantitative meta-analyses have estimated consistently and significantly increased risks for multiple outcomes associated with dampness or mold, including OR ranges of 1.30–1.75.

Substantial increases in a number of important respiratory health outcomes, including a 50% increase in current asthma, are associated with dampness-related risk factors in residences (Fisk et al. 2007). These estimates, based on limited data, broad lumping of diverse risk factors, and multiple unverified assumptions, should be interpreted cautiously; however, they indicate that dampness-related risk factors may contribute substantially, but preventably, to the burden of respiratory disease.

In agreement with the IOM report (2004), we consider that there is not sufficient epidemiologic evidence of a causal relationship for any of the reviewed health outcomes, although for asthma exacerbation in children we consider the evidence strongly suggestive of causality by dampness-related agents. Although it is plausible that microbial exposures may play a causal role, specific causative agents have not been established. In fact, limited and inconsistent evidence suggests that moderate exposures to certain microbial agents, especially at early ages, may prevent allergies and allergic asthma.

Based on available evidence, the presence of dampness, water damage, visible mold or mold odors or a history of water damage provides more reliable indicators of dampness- or mold-related health risks than do current quantitative microbiologic assessments. As reduction of indoor dampness and mold is likely to have benefits for respiratory and allergic health of occupants, this level of knowledge should guide practical prevention and remediation now. Still, available research does not yet indicate the amount of water damage, mold, or mold odor meriting concern nor document the relative magnitude of health benefits from different environmental remediations.

Although Williamson et al. (1997) published findings of strong, dose-related associations of asthma severity with systematic moisture measurements in walls 13 years ago, research use of quantified dampness metrics has not been reported since. Future research, generally, should develop objective metrics for dampness-related and microbial (or nonmicrobial) risk factors that predict health effects. This will help in identifying specific causal dampness-related agents and characterizing exposure–response relationships.

Challenges to progress include the wide variety of currently plausible microorganisms (fungi, bacteria, amoebae/protozoans) and microbial components and products eligible to be causal factors; the potentially nonmonotonic effects of some of these components (e.g., glucans and endotoxin); the potential synergistic actions of some organisms, including actinomycetes and amoebae; the possible involvement of nonbiological, chemical agents released from damp indoor materials; and the modification of microbial effects by human age at exposure or by genetic or other host susceptibility factors. However, although their effectiveness may ultimately be improved, prevention and remediation actions to reduce indoor dampness are important and urgently needed in a large proportion of our building stock. These measures are likely to significantly reduce the current global burden of respiratory and allergic disease.

## Figures and Tables

**Table 1:** Total numbers of published studies on health effects: those cited by the IOM review (IOM 2004) and those identified later and included in this review, plus summary^a^ of findings only for qualitative^b^ assessments of dampness or mold.

		Total number of studies		Summary of qualitative assessments of dampness or mold^b^	
Health outcome category	Study design	IOM review	New	OR range^c^	Proportion of total estimates showing any positive association with D/M^d^
Asthma development	Prospective	2	4	0.65–7.08	7/9
	Retrospective	6	2	0.63–4.12	29/38
	Cross-sectional	0	3	1.6–2.2	2/2

Asthma symptoms in asthmatic people (exacerbation)	Intervention	0	3	No ORs	22/22
	Prospective	0	1	3.8–7.6	2/2
	Retrospective	5	0	1.5–4.9	7/7
	Cross-sectional	18	4	1.0–7.6	45/47

Ever-diagnosed asthma	Prospective	—	2	1.2–1.3	2/2
	Cross-sectional	—	18	0.6–2.6	31/33

Current asthma	Prospective	—	1	No qual	No qual
	Cross-sectional	—	25	0.3–13.0	60/64

Dyspnea	Intervention	0	1	No ORs	2/2
	Cross-sectional	4	11	0.4–9.4	56/67

Wheeze	Intervention	0	1	No ORs	7/8
	Prospective	0	12	0.68–6.17	35/37
	Retrospective	1	1	1.5–2.8	9/9
	Cross-sectional	19	41	0.44–8.67	151/164

Bronchitis	Prospective	—	1	0.7–3.8	4/5
	Cross-sectional	—	11	1.2–2.4	19/19

Altered lung function	Intervention	—	2	No ORs	6/6
	Prospective	—	2	No ORs	7/13
	Retrospective	—	1	No ORs	4/8
	Cross-sectional	—	6	No ORs	8/9

Cough	Prospective	0	2	0.54–2.14	7/9
	Retrospective	1	0	1.18–1.90	4/4
	Cross-sectional	20	26	0.21–5.74	140/147

Respiratory infections and otitis media	Prospective	—	5	0.45–5.1	14/24
	Cross-sectional	—	13	0.48–3.14	30/37

Common cold	Prospective	—	1	0.6–1.8	4/9
	Cross-sectional	—	5	0.98–1.7	13/14

Eczema	Prospective	—	2	1.2–2.9	3/3
	Cross-sectional	—	4	0.3–1.9	13/15

Allergy/atopy (excluding allergic rhinitis and eczema)	Prospective	—	7	0.6–2.4	9/12
	Cross-sectional	—	15	1.1–1.9	15/19^e^

Allergic rhinitis	Prospective	—	2	1.2–3.2	5/5
	Cross-sectional	—	3	0.7–3.5	7/8

Upper respiratory tract symptoms (including allergic rhinitis)	Intervention	0	1	No ORs	5/6
	Prospective	0	5	1.03–3.2	11/11
	Retrospective	0	1	1.0–1.3	1/2
	Cross-sectional	14	20	0.37–5.92	107/122

Other respiratory	Prospective	—	5	1.03–1.06	4/4
	Cross-sectional	—	13	0.45–2.4	11/14

Total studies		45^f^	103^f^		

Abbreviations: —, outcome not included in review; D/M, dampness or mold; no qual, no qualitative exposure assessments in article.

a For details regarding the studies in this table, see Supplemental Material, Tables A1.1–A1.6 and A2.1–A2.6 (doi:10.1289/ehp.1002410).

b Findings for quantified microbiologic factors omitted.

c Includes all reported ratio estimates of effect: ORs, RRs, IRRs.

d Proportion of findings with ORs, RRs, or IRRS > 1.0 (or < 1.0 for removal of D/M) or nonratio estimates, such as linear coefficients, greater/less than 0 or 1 as appropriate.

e Although all reported ORs/RRs/IRRs exceeded 1.0, other types of estimates were not consistent.

f Totals are less than the sum of the numbers above, as each study may report multiple findings.

**Table 2:** Summary estimates from three meta-analyses on residential D/M and health.

	Subject groups	OR (95% CI)		
		Fisk et al. 2007^a^	Fisk et al. 2010^a^	Antova et al. 2008^b^
Upper respiratory tract symptoms	All	1.70 (1.44–2.00)		

Cough	All	1.67 (1.49–1.86)		
	Adults	1.52 (1.18–1.96)		1.30 (1.22–1.39)^c^
	Children	1.75 (1.56–1.96)		1.50 (1.31–1.73)^d^

Wheeze	All	1.50 (1.38–1.64)		
	Adults	1.39 (1.04–1.85)		1.43 (1.36–1.49)^e^
	Children	1.53 (1.39–1.68)		1.49 (1.28–1.74)^f^

Current asthma	All	1.56 (1.30–1.86)		

Ever-diagnosed asthma	All	1.37 (1.23–1.53)		
	Children			1.35 (1.20–1.51)

Asthma development	All	1.34 (0.86–2.10)		

Bronchitis	All		1.45 (1.32–1.59)	
	Children			1.38 (1.28–1.47)

Respiratory infections	All		1.44 (1.31–1.59)	
	Adults		1.49 (1.14–1.95)	
	Children		1.48 (1.33–1.65)	

Respiratory infections^g^	All		1.50 (1.32–1.70)	

Sensitivity to inhaled antigens	Children			1.33 (1.23–1.44)

Hay fever	Children			1.35 (1.18–1.53)

a Based on all eligible published studies at the time, ranging from 4 to 22 studies for each outcome; all risk factors of visible mold, visible water damage, mold odor, and various combinations of these were included together.

b Based on a total of 12 studies in 12 countries, including over 57,000 children: 10 studies of any visible mold, 1 study of any visible mold in last 12 months, and 1 study of any visible mold in child’s bedroom.

c Nocturnal dry cough.

d Morning cough.

e Wheeze in the last 12 months.

f Woken by wheeze.

g Including lower respiratory infections, tonsillitis, sinusitis, otitis, and pharyngitis, but excluding nonspecific upper respiratory infections.

**Table 3:** Level of confidence for associations between indoor dampness or dampness-related agents^a^ and health outcomes, based on epidemiologic evidence.^b^

Updated conclusion^a^	Outcome	Additional evidence^c^	Prior IOM conclusion
Sufficient evidence of a causal relationship	(None)	(None)	(None)
Sufficient evidence of association	Asthma exacerbation	More studies of strong design (strongly suggestive of causation)	Sufficient evidence of association
	Cough^d^	Many new studies, some of strong design	Sufficient evidence of association
	Wheeze^d^	Many new studies, many of strong design	Sufficient evidence of association
	Upper respirator*y* tract symptoms^d^	Many new studies, some of strong design	Sufficient evidence of association
	Asthma development^e^	More studies of strong design	Limited or suggestive evidence of association
	Dyspnea^e^	More studies	Limited or suggestive evidence of association
	Current asthma^d^,^e^	Initial evaluation	Not evaluated
	Ever-diagnosed asthma^d^,^e^	Initial evaluation	Not evaluated
	Respiratory infections^e^	Initial evaluation	Not evaluated
	Bronchitis^d^,^e^	Initial evaluation	Not evaluated
	Allergic rhinitis^d^,^e^	Initial evaluation	Not evaluated
	Eczema^d^,^e^	Initial evaluation	Not evaluated
Limited or suggestive evidence of association	Common cold^e^	Initial evaluation	Not evaluated
	Allergy/atopy^d^,^e^	Initial evaluation	Not evaluated
Inadequate or insufficient evidence to determine whether an association exists	Altered lung function	Initial evaluation	Not evaluated
	Hypersensitivity pneumonitis	(None)	(Association based on clinical evidence)

a Based on evidence of visible water damage, visible mold, mold odor, or similar related factors.

b Association between hypersensitivity pneumonitis in susceptible individuals and the presence of mold or other agents is documented by clinical evidence (IOM 2004).

c Studies of stronger design include experimental, cohort, or case–control designs.

d Statistically significant elevation of risk identified in a quantitative meta-analysis.

e Conclusion changed from IOM conclusion.

**Table 4:** Measured indoor microbiologic factors with suggestive positive or negative associations^a^ with specific respiratory or allergic health effects in building occupants.^b^

Measured microbiologic factors	Specific health outcomes	Findings with suggestive positive associations	Findings with suggestive negative associations	No. of studies	Range of ORs	References
Ergosterol in dust, higher levels	Current asthma	5 of 6 (83%)		3	0.92–4-fold	Dharmage et al. 2001; Matheson et al. 2005; Park et al. 2008
Endotoxin in dust, higher levels	Wheeze	20 of 25 (80%)		14	0.67–2.8	Iossifova et al. 2007, 2009; Park et al. 2001, 2006; Zhao et al. 2008; Schram-Bijkerk et al. 2005; Bolte et al. 2003; Campo et al. 2006; Douwes et al. 2006; Gehring et al. 2008; Gillespie et al. 2006; Litonjua et al. 2002; Perzanowski et al. 2006
(1→3)-β-d-glucans in dust, medium levels	Wheeze	7 of 8 (88%)		3	0.89–6.05	Douwes et al. 2006; Iossifova et al. 2007, 2009
(1→3)-β-d-glucans in dust, highest levels	Wheeze		10 of 11 (91%)	4	0–1.25	Douwes et al. 2006; Iossifova et al. 2007, 2009; Schram-Bijkerk et al. 2005

a A suggestive association required, among reported findings on associations between a specific measured indoor microbiologic factor and a specific respiratory or allergic health outcome, at least 80% consistency of estimates either ≤ 1.0 or > 1.0, among at least five estimates available from three or more studies. This assessment did not consider magnitude of effects, precision, statistical significance, study design, or age of subjects.

b Measured microbiologic factors with inadequate or insufficient evidence to determine whether an association exists with any specific health outcome are listed in Supplemental Material, Table A3.1 (doi:10.1289/ehp.1002410).
